# The prognostic value of the serum ferritin in a southern Brazilian cohort of patients with Gaucher disease

**DOI:** 10.1590/1678-4685-GMB-2015-0125

**Published:** 2016

**Authors:** Tiago Koppe, Divair Doneda, Marina Siebert, Livia Paskulin, Matheus Camargo, Kristiane Michelin Tirelli, Filippo Vairo, Liane Daudt, Ida Vanessa D. Schwartz

**Affiliations:** 1 Departamento de Genética, Universidade Federal do Rio Grande do Sul (UFRGS), Porto Alegre, RS, Brazil; 2 Programa de Pós-Graduação em Genética e Biologia Molecular, Universidade Federal do Rio Grande do Sul (UFRGS), Porto Alegre, RS, Brazil; 3 Laboratório de Técnica Dietética, Faculdade de Medicina, Universidade Federal do Rio Grande do Sul (UFRGS), Porto Alegre, RS, Brazil; 4 Serviço de Genética Médica, Hospital de Clínicas de Porto Alegre (HCPA), Porto Alegre, RS, Brazil; 5 Serviço de Hematologia Clínica, Hospital de Clínicas de Porto Alegre (HCPA), Porto Alegre, RS, Brazil; 6 Basic Research and Advanced Investigations in Neurosciences (BRAIN) Laboratory, Hospital de Clínicas (HCPA), Porto Alegre, RS, Brazil; 7 Unidade de Análises Moleculares e de Proteínas (UAMP), Centro de Pesquisa Experimental, Hospital de Clínicas de Porto Alegre (HCPA), Porto Alegre, RS, Brazil

**Keywords:** ferritin, biomarkers, Gaucher disease, iron metabolism

## Abstract

The clinical utility of serum ferritin as a biomarker of disease severity and prognosis in Gaucher disease (GD) is still debated. Here, we aimed to evaluate ferritin and its relation to clinicolaboratory parameters of GD patients seen at the Reference Center for Gaucher Disease of Rio Grande do Sul, Brazil, so as to gather evidence on the utility of ferritin as a biomarker of this condition. A retrospective chart review was performed collecting pre-and posttreatment data from GD patients. Eighteen patients with ferritin levels available before and after treatment were included in the study. Nine of these participants were males, and seventeen had type I GD. All patients were given either enzyme replacement (n = 16) or substrate reduction therapy (n = 2), and ferritin was found to decrease from 756 [318-1441] ng/mL at baseline to 521 [227-626] ng/mL (p=0.025) after 28.8 month soft treatment. Serum ferritin levels did not correlate with measures of disease severity, but showed an association with age at onset of treatment (ρ= 0.880; n = 18; p < 0.001). In conclusion, although serum ferritin did not correlate with disease severity, after a median 28.8 months of treatment, clinical outcomes had clearly improved, and ferritin levels had decreased.

## Introduction

Gaucher disease (GD) (OMIM #230800) is a metabolic disorder caused by a deficiency in lysosomal glucocerebrosidase activity (EC 3.2.1.45) due to mutations in the*GBA1* gene (Grabowski *et al.*, 2014). The worldwide prevalence of GD is about 1:40,000-200,000 (Poorthuis *et al.*, 1999; Pinto *et al.*, 2004), and its clinical manifestations include multisystem organomegaly, pancytopenia and bone complications (Pastores and Hughes, 2000; Grabowski *et al.*, 2014). Although the mechanisms by which*GBA1* gene mutations influence GD phenotype remain largely unknown, the main pathogenesis of this disorder is believed to be the lysosomal accumulation of glucocerebroside in mononuclear phagocytes. This process triggers the expression of cytokines and other inflammatory molecules which, in turn, engage other immune cells and ultimately lead to a systemic immune response (Barak *et al.*, 1999; Cox, 2001; Boven*et al.*, 2004; Liu*et al.*, 2012; Pandey and Grabowski, 2013; Vairo *et al.*, 2013; Thomas *et al.*, 2014). Several circulating molecules thought to be secreted by these lipidladen macrophages have been studied as biological markers of disease burden and/or response to enzyme replacement therapy (ERT) (Aerts et al., 2005; Cox, 2006). In this re- gard, although lyso-Gb1 has recently emerged as a promising specific biomarker of GD (Fuller *et al.*, 2015), the most frequent and consistently altered molecules in GD have been found to be the following: chitotriosidase (EC 3.2.1.14), angiotensin converting enzyme (EC 3.4.15.1), CCL18 (P55774), tartarate-resistant acid phosphatase (EC3.1.3.2) and serum ferritin (SF) (P02792 and P02794) (Aronson, 2005; Boot *et al.*, 2009; Hughes and Pastores, 2013; Thomas *et al.*, 2013, 2014). Over the past few years, several studies have described hyperferritinemia in GD patients and demonstrated its reduction after the initiation of treatment. The first study to report hyperferritinemia over the course of GD was published by Morgan *et al.* (1983), and since then these findings have been replicated in different cohorts worldwide (Poll*et al.*, 2002; Grosbois *et al.*, 2009; Saadi *et al.*, 2010; Stein *et al.*, 2010; Stirnemann *et al.*, 2010; Stirnemann *et al.*, 2011; Mekinian *et al.*, 2012). The reduction in SF exhibited by patients with GD undergoing ERT could be indicative of a positive clinical outcome (Stirnemann *et al.*, 2010; Vigan *et al.*, 2014), although no studies to date have identified a clear association between SF levels and GD severity. In Brazilian patients, these findings have yet to be replicated. As such, the aim of this study was to evaluate the clinical use of SF as a biomarker of GD severity and/or response to treatment in a Brazilian cohort of GD patients.

## Material and Methods

A retrospective chart review of patients with GD treated at the GD Reference Center of Rio Grande do Sul, Brazil, was performed. An extensive database of patients with GD was analyzed, and all those who had at least two measurements of SF levels -one before and the other after the onset of GD therapy (*i.e.*, ERT or substrate reduction therapy -SRT) – were included in the study.

Pre-treatment SF levels were defined as the most recent measurements available in the database prior to the onset of treatment, and post-treatment SF was evaluated based on the most recent measurement available for each patient in the dataset. Hyperferritinemia was defined as SF > 322 ng/mL in males and > 291 ng/mL in females. All other variables were matched to SF levels by date to determine their status as pre-or post-treatment measures. In the referral center, the diagnosis of GD is confirmed in all cases by reduced glucocerebrosidase activity (*i.e.*, < 10% of normal) in leukocytes. All patients received standard care, and had their clinical history, physical examinations, lab tests and medical imaging results recorded during routine medical assessments.

Asthenia, abdominal pain, bone involvement, bleeding, splenomegaly and hepatomegaly were evaluated based on clinical assessments or ancillary tests recommended by current guidelines. SSI (Severity Score Index) was calculated on every medical visit as described in Zimran *et al.*(1989). Quantitative variables were expressed as median [25th-75th percentiles], and categorical variables were displayed as absolute frequencies. Non-parametric tests were used to compare pre-and post-treatment variables and assess treatment effects. Two-tailed Wilcoxon signed-rank tests were used to analyze quantitative variables, while McNemar tests were involved in the evaluation of categorical data. Additionally, Spearman correlation analyses were carried out to assess the relationship between quantitative variables and SF. All tests were performed using SPSS 18 (IBM^®^) and statistical significance was considered when *p* < 0.05.

This study was approved by the Research Ethics Committee of the Hospital de Clínicas de Porto Alegre, Brazil.

## Results

Eighteen patients with a diagnosis of GD met inclusion criteria for this study (male = 9, females = 9; GD type I = 17; GD type III = 1). Eleven patients had a N370S/L444P genotype, three were N370S/N370S, two were N370S/RecNciI, one was E349K/S366N and one, L444P/L444P. Median age at diagnosis and treatment onset was 39.5 years [15.7-51.2] and 41.1 years [24.6-52.9], respectively. Three patients had undergone splenectomy, one had cirrhosis and none had hepatocellular carcinoma. Patients had no history of blood transfusions, although three female patients received a short course of iron supplementation over the follow-up and were excluded from the analysis of serum iron levels and transferrin saturation. At inclusion, sixteen patients were on ERT (velaglucerase alpha = 1; taliglucerase alpha = 2; imiglucerase = 13), and received a mean dosage of 21.56 ± 12.21 UI/kg/inf every 2 weeks. Additionally, two patients were receiving SRT (eliglustat = 1; miglustat = 1). None of the 18 participants had any other biochemical evidence of iron overload, such as high transferrin saturation.

A consistent effect of therapy was observed in comparison between pre-and post-treatment clinical and laboratory data (Table 1). Treatment was also associated with a significant reduction in SF levels (Table 1). Prior to treatment, 14 patients (77.8%; 9/9 males and 5/9 females) exhibited hyperferritinemia, including all three splenectomized patients. All females without hyperferritinemia were younger than 29 years. In the post-treatment period, 12 patients (66.7%; 7/9 males and 5/9 females) presented hyperferritinemia, including all three splenectomized patients. The subgroup of the hyperferritinemic patients that normalized SF and the subgroup that did not normalize did not differ regarding all the other analyzed variables (data not shown). Correlations between SF and hemoglobin, platelets, alanine aminotransferase, lactate dehydrogenase, chitotriosidase, and SSI were not statistically significant (data not shown). However, pre-treatment SF levels were associated with age at onset of the treatment (ρ= 0.880; *n* = 18;*p* < 0.001) and Body Mass Index (BMI) (ρ= 0.713;*n* = 14; *p* < 0.005).

**Table 1:** Hyperferritinemia in patients with Gaucher disease: pre- and post-treatment data.

	Pre-treatment (n = 18)	Post-treatment (n = 18)	*p*
Clinical findings:			
-Abdominal pain	11	5	0.070
**-Bleeding** *	**10**	**2**	**0.008**
-Bone involvement^1^	14	12	0.500
-Splenomegaly^2^	14/n = 15	11/n = 15	0.250
-Hepatomegaly	11	6	0.125
-BMI^3^(kg/m^2^)	23.8 [20.9-27.7]	24.6 [21.6-28.1]	0.170
**Hemoglobin** * **(g/dL)**	**12.6 [10.1-13.9]**	**14 [11.6-15]**	**0.005**
**Leukocytes (x103** /μL)*	**4.15 [2.93-6.8]**	**4.9 [4.5-7]**	**0.050**
**Platelets** * **(x103** /μL)	**79 [63.7-146.7]**	**145.5 [116-171]**	**0.002**
ALT (IU/L)	22.5 [15-35.7]	27 [18-43]/n = 17	0.093
LDH (IU/L)	317 [246-360]/n = 15	296 [238-368]/n = 17	0.778
**Chitotriosidase** * **(x103) nmol/h/mL)**	**14 [7.9-14.7]**	**3.7 [1.7-5.5]**	**0.002**
**SSI** 4 *	**7 [4-12]/n = 15**	**2 [1-5.5]/n = 17**	**0.001**
BMB score^5^	8.5 [4.5-12]/n = 14	7 [4.25-10]/n = 12	0.180
**Serum ferritin (ng/mL)** *			
**-Total** *	**756 [318-1441]**	**521 [227-626]**	**0.025**
**-Male** *	**811 [614-1544]/n = 9**	**528 [369-653]/n = 9**	**0.038**
-Female	328 [190-1174]/n = 9	362 [123-624]/n = 9	0.374
Transferrin saturation (%)	19 [14-32]/n = 8	28 [23-34]/n = 13	0.138
Serum iron (μg/dL)	56 [46-89]/n = 10	91 [72-99]/n = 13	0.176

Values expressed as median [25th-75th per centiles] or absolute count, unless otherwise specified;

* *p* <0.05 after McNemar or Wilcoxon signed-rank test; Reference values: serum ferritin (males: 22-322 ng/mL and females: 10-291 ng/mL); transferrin saturation (25-50%) and serum iron (50-170 μg/dL).

1 Bone involvement was considered whenever patients mentioned pain/pathological fractures without any other attributable cause, or in the presence of imaging evidence of bone disease (*e.g.*, osteopenia on bone densitometry).

2 Three patients had undergone splenectomy.

3 Body Mass Index (Kg/m): < 18.5 – underweight; 18.5-24.9 – normal weight; 25-29.9 – overweight and > 30 – obesity

4 Clinical Severity Score Index: 0-9 – mild; 10-19 – moderate and > 20 – severe.

5 Bone Marrow Burden score: 0-4 – mild; 5-8 – moderate and 9-16 – severe.

## Discussion

This study aimed to investigate the clinical use of SF as a biomarker of disease severity and/or response to treatment in GD. Our results showed that hyperferritinemia is a common occurrence in GD, especially in untreated, older male patients with a higher BMI. Our results are also in agreement with findings obtained both from general and GD populations. SF levels are known to be influenced by sex and age, so that reference values differ by age and gender, in addition to population characteristics, such as ethnicity and presence of other medical comorbidities (Luxton *et al.*, 1977; Vicente *et al.*, 1990; Custer *et al.*, 1995; Koziol *et al.*, 2001; Pan and Jackson, 2008; Ferraro *et al.*, 2012). Growing evidence has shown that SF is associated with BMI, obesity and insulin resistance, and nonalcoholic fatty liver disease (Guglielmi*et al.*, 2015). In addition, several studies have found that women, regardless of age and presence of medical comorbidities, always tend to have lower SF values than men. Additionally, premenopausal women have dramatically lower SF levels than postmenopausal females. The peaking of SF levels in menopause has not been associated with transferrin saturation (Koziol *et al.*, 2001), but has been found to be related to hepcidin, the central peptide in iron metabolism regulation, whose levels are very strongly correlated with those of SF (Galesloot *et al.*, 2011). The cutoff for hyperferritinemia varies across contexts. In our sample, only 55% of the women were labeled as hyperferritinemic according to the cutoff points provided by the laboratory where the analyses were performed. However, when the female cutoff values suggested by the American Association for the Study of Liver Diseases (15150 ng/mL) (Bacon *et al.*, 2011) were applied to the sample, the percentage of females categorized as hyperferritinemic was found to be higher (data not shown).

The magnitude of the increase in SF levels in patients with GD was very similar to that observed in a previous study (median 756 *vs.* 739 ng/mL, respectively) (Mekinian *et al.*, 2012), but lower than that reported by Stein *et al.* (2010), who found a 3.7-fold elevation above the upper limit of normality. These differences and similarities may be attributable to mean sample age, which was very similar between the present study and that performed by Mekinian *et al.* (2012), but much lower in our sample than in the participants recruited by Stein *et al.* (2010).

Our data did not support the use of SF as a biomarker of GD severity. Previous studies have shown the same findings, suggesting that SF levels may not be influenced by disease severity *per se*, but by the number of years of disease activity (*i.e.*, older individuals). Low SF is pathognomonic of iron deficiency and can be used to detect this condition with almost 100% specificity. However, high SF levels are not necessarily indicative of iron overload. Therefore, a transferrin saturation cutoff value > 45% has been suggested as a good predictor of iron overload (Gilles, 2013). In inflammatory conditions, such as GD, iron becomes unavailable for erythropoiesis, despite adequate iron storage levels. This phenomenon is called functional iron deficiency and is characteristic of the anemia of chronic disorders. So, hyperferritinemia may exist in the presence or in the absence of iron overload, and recent studies have demonstrated macrophage iron accumulation in anemia of chronic disease (King and Weiss, 2014). In this regard, iron overload in inflammatory cells has also been identified in conditions such as Parkinson's disease, multiple sclerosis and other neurodegenerative disorders using noninvasive imaging methods to quantify iron levels in different tissues (Stoll and Bendszus, 2009; Böttcher *et al.*, 2013; Mehta *et al.*, 2013). The analysis of participant iron profiles showed that transferrin saturation and serum iron levels also appeared to increase after treatment, although this change was not statistically significant in our sample. Since iron deficiency and iron deficiency anemia is far more frequent in the Brazilian population than in people from developed countries and this tendency is reflected in Brazilian GD cohorts (Sobreira*et al.*, 2007), this lack of statistical significance could reflect a poor-iron diet. Nonetheless, taken together these findings may suggest that patients under treatment may have higher concentrations of iron available for erythropoiesis.

In conclusion, SF does not appear to be a useful biomarker of disease severity in GD, but can be used as a biomarker of diagnosis and response to treatment. Treatment also seemed to increase serum iron and transferrin saturation levels, increasing the amount of iron available for erythropoiesis. These findings need to be further evaluated in order to clarify the relation between SF and iron metabolism in GD patients.
